# Thyroid function and age-related decline in kidney function in older Chinese adults: a cross-sectional study

**DOI:** 10.1186/s12877-022-02904-z

**Published:** 2022-03-17

**Authors:** Lu Wei, Yun Bai, Yu Zhang, Zhenzhu Yong, Bei Zhu, Qun Zhang, Weihong Zhao

**Affiliations:** 1 Division of Nephrology, Department of Geriatrics, The First Affiliated Hospital of Nanjing Medical University, No. 300 Guangzhou Road, Jiangsu, 210029 China; 2 Health Management Center, The First Affiliated Hospital of Nanjing Medical University, Nanjing, China

**Keywords:** Thyroid function, Kidney function, Glomerular filtration rate, Aging

## Abstract

**Background:**

Thyroid function may be a factor affecting kidney function in the general population. Kidney and thyroid function vary with age; therefore, the association between thyroid function and decreased kidney function in older adults may be different from that in younger adults and remains controversial. This study aimed to estimate the association between normal-range thyroid function and age-related decline in kidney function in older Chinese adults.

**Methods:**

A total of 15,653 adults, of whom 23.2% (*N* = 3624) were older adults (age≧65 years), were collected at the Health Management Center of the First Affiliated Hospital of Nanjing Medical University from January 2018 to January 2020. Basic demographic information was collected by a physician-administered questionnaire. The estimated glomerular filtration rate (eGFR) was calculated using the CKD-EPI formula. Trends in thyroid function with age were shown by means of free triiodothyronine (FT3), free thyroxine (FT4), and thyroid-stimulating hormone (TSH) in subgroups every ten years. The association between kidney function and thyroid function was estimated by multiple linear regression using β value and by multivariable logistic regression models using odds ratios (OR) after adjusting for age, gender, body mass index, and serum urine acid.

**Results:**

In the older population, TSH tended to increase with age and FT3 tended to decrease, whereas FT4 was relatively stable. eGFR decreased significantly with increasing TSH (β = -0.081) and decreasing FT3 (β = 0.083) concentrations. Compared with those in the lowest quartile of FT3 (3.10–4.47 pmol/L), the prevalence of eGFR < 75 ml/min/1.73m^2^ decreased significantly by 22.0% for those with FT3 of 4.47–4.81 pmol/L, 27.6% for those with FT3 of 4.82–5.20 pmol/L, and 34.9% for those with FT3 of 5.21–6.8 pmol/L in older individuals (*P* for trend < .001). The OR was 1.315 (*P*: 0.025) in subjects with high-normal TSH, using low-normal TSH as a reference. The prevalence of reduced kidney function was not significantly associated with FT4 within the reference range. Similar results were found in association between the prevalence of eGFR < 60 ml/min/1.73m^2^ and thyroid function.

**Conclusions:**

This study demonstrated a significant association between kidney function and thyroid function, particularly FT3, in the older population. Clinicians may need to pay more attention to the assessment and follow-up of kidney function in older individuals with low-normal FT3 and high-normal TSH.

**Supplementary Information:**

The online version contains supplementary material available at 10.1186/s12877-022-02904-z.

## Background

Kidney function declines with age, mainly in the form of decreased glomerular filtration rate (GFR), accompanied by decreased tubular function and structural changes [1]. These impairments caused by aging make older adults more susceptible to acute kidney injury and increase the propensity to subsequent progressive chronic kidney disease (CKD) [2]. The decline in kidney function with advancing age is accompanied by other age-related comorbidities whose presence may affect kidney function [3, 4].

In the general population, previous studies have elucidated, nationally and internationally, that thyroid function is associated with alterations in kidney function [5, 6]. Indicators of thyroid function vary with aging [7]. Therefore, the association between thyroid function and decreased kidney function in older adults may differ from that in younger adults [8]. Several studies have shown that thyroid dysfunction is associated with estimated GFR (eGFR) and the likelihood of prevalent CKD in older adults, but which indicator of thyroid function is more relevant to kidney function and the association between them remains controversial [9, 10]. For instance, Meuwese et al. clarified that free thyroxine (FT4) is a positive influence on GFR in older adults [11], whereas another study suggested that elevated FT4 was associated with an increased risk of CKD in the Chinese general population [12]. No data to date investigates the association between normal-range thyroid function and age-related kidney function among general older adults in China.

Therefore, this study was conducted to clarify the association between indicators of thyroid function within the reference range and kidney function in the older Chinese population.

## Methods

### Participants

This study was a retrospective cross-sectional study. A total of 15,653 Chinese adults from January 2018 to January 2020 were collected at the Health Management Center of the First Affiliated Hospital of Nanjing Medical University. All participants underwent a standardized physical and neuropsychological examinations. Inclusion criteria were as follows: (1) age≧18 years, (2) body mass index (BMI): 18.5–28 kg/m^2^, (3) indicators of thyroid function (free triiodothyronine (FT3), free thyroxine (FT4), and thyroid-stimulating hormone (TSH)) within the reference interval, and (4) other indicators: blood pressure (BP), urine acid (UA), blood glucose (BG), alanine transaminase (ALT), and low-density lipoprotein cholesterol (LDL-C) in relatively stable state according to guidelines based on the Chinese population. Individuals with the following conditions were excluded: (1) missing serum creatinine (SCr), thyroid hormone values or demographic variables; (2) diffuse echogenic changes, enlarged thyroid, and absent thyroid by thyroid ultrasound; (3) proteinuria, eGFR < 60 ml/min/1.73m^2^ in younger adults or eGFR < 45 ml/min/1.73m^2^ in older adults; (4) taking medications that affect thyroid function, such as amiodarone, lithium, iodine, levothyroxine, and antithyroid drugs; (5) history of malignant tumors, kidney disease and other systemic diseases; (6) pregnant status, acute onset or serious systemic diseases. The ethical review committee of the First Affiliated Hospital of Nanjing Medical University approved this study (2018-SR-181).

### Laboratory measures

Basic demographic information (age, gender, past medical history, and related medications) was collected by a physician-administered questionnaire. Questionnaires were assessed for incomplete or inconsistent answers by nurses and re-evaluated by our physicians. BMI was calculated by dividing the measured weight in kilogram by the square of height in meters.

Blood samples were collected in the morning after an overnight fast of at least 8 h. After separation, plasma/serum samples were stored at 4℃ in refrigerated containers and sent to the laboratory. SCr was measured by an enzymatic method (Shanghai Kehua Dongling Diagnostic Products Co., Ltd, China). The above samples were strictly following the rules of operation and assayed in Olympus AU5800 automatic biochemical instrument (Beckman Coulter, Inc., USA). The CLIA immunoassay was used to measure TSH (reference range: 0.27–4.2 mIU/L) levels. FT4 (reference range: 12–22 pmol/L) and FT3 (reference range: 3.1–6.8 pmol/L) were measured by microparticle enzyme immunoassays.

### Assessment of kidney function

eGFR was calculated using the 2009 Chronic Kidney Disease Epidemiology Collaboration (CKD-EPI) equation. For females, if SCr is ≤ 0.7 mg/dl, eGFR equals to 144 × (Cr/0.7) ^−0.329^ × (0.993) ^age^, and if SCr is > 0.7 mg/dl, eGFR equals to 144 × (Cr/0.7) ^−1.209^ × (0.993) ^age^. For males, if SCr is ≤ 0.9 mg/dl, eGFR equals to 141 × (Cr/0.9) ^−0.411^ × (0.993) ^age^, and if SCr is > 0.9 mg/dl, eGFR equals to 141 × (Cr/0.9) ^−1.209^ × (0.993) ^age^.

### Statistical analysis

Baseline characteristics were presented as mean ± SD for continuous variables and as proportions for categorical variables. T-test and Chi-square test were used to determine statistical differences between the younger and older adults. To investigate the trend of thyroid function and eGFR with aging, we divided the total population into seven subgroups according to age and calculate the mean of above indicators in each subgroup. The differences between groups and pairwise comparisons were analyzed using one-way ANOVA and Duncan’s multiple range tests, respectively. The association between thyroid function and eGFR was assessed by multiple linear regression analysis. Using the lowest quartile range of FT3, FT4 or TSH as the reference, we performed multivariable logistic regression analysis to estimate the risk of reduced kidney function under different thyroid functions. Primary logistic regression analyses were performed with adjustment for age, gender, BMI, and UA, and secondary analyses were performed with adjustment for above variables, hypertension, and diabetes.

Statistical analyses were performed using IBM® SPSS® statistics version 26.0. (IBM Corporation, New York, USA). Figures were created by Prism 8 (GraphPad, 1992). Two-tailed *P* value of < 0.05 was statistically significant.

## Results

The Characteristics of the total participants were shown in Table 1. The mean age of the total population was 54.36 years, of which 23.2% (*N* = 3624) were older adults older than or equal to 65 years old. Compared to younger adults, the proportion of the female and LDL-C levels were significantly lower in older adults, whereas BMI, SBP, UA, and BG were significantly higher. The prevalence of eGFR < 75 ml/min/1.73m^2^ was 7.5% (*N* = 1177) in total participants, 2.3% (*N* = 273) in younger adults, and 24.9% (*N* = 904) in older adults. The prevalence of eGFR < 60 ml/min/1.73m^2^ was 5.5% (*N* = 200) in older population.

**Table 1:** Characteristics of the study populations

	Total	Younger Adults (< 65ys)	Older Adults (≧65ys)
*N* (%)	15,653	12,029 (76.8%)	3624 (23.2%)
Female (*N*, %)	6063 (38.7%)	4921 (40.9%)	1142 (31.5%) ^*^
Age (ys)	54.36 (13.98)	48.66 (10.07)	73.28 (6.43) ^*^
BMI (ml/m^2^)	23.62 (2.33)	23.53 (2.35)	23.93 (2.25) ^*^
SBP (mmHg)	128.7 (18.20)	124.48 (16.15)	142.67 (17.64) ^*^
DBP (mmHg)	76.98 (10.84)	76.83 (10.88)	77.5 (10.69)
Hypertension (*N*, %)	5835 (37.3%)	3277 (27.2%)	2558 (70.6%) ^*^
Diabetes (*N*, %)	1469 (9.4%)	781 (6.5%)	688 (19.0%) ^*^
BUN (mmol/L)	5.13 (1.20)	5.04 (1.15)	5.44 (1.28) ^*^
SCr (mg/dL)	0.80 (0.16)	0.78 (0.15)	0.84 (0.17) ^*^
UA (υmol/L)	338.39 (80.22)	335.5 (80.47)	348.0 (78.64) ^*^
LDL-C (mmol/L)	3.29 (0.8)	3.34 (0.79)	3.11 (0.84) ^*^
BG (mmol/L)	5.55 (1.07)	5.42 (0.95)	6.01 (1.31) ^*^
FT3 (pmol/L)	4.84 (0.56)	4.89 (0.56)	4.66 (0.51) ^*^
FT4 (pmol/L)	17.18 (1.97)	17.21 (1.95)	17.09 (2.05) ^*^
TSH (mIU/L)	2.28 (1.21)	2.23 (1.07)	2.44 (1.59) ^*^
eGFR (ml/min/1.73m^2^)	96.79 (14.52)	101.33 (12.12)	81.71 (11.24) ^*^
> 90 ml/min/1.73m^2^	11,074 (70.7%)	10,116 (84.1%)	958 (26.4%)
75–90 ml/min/1.73m^2^	3402 (21.7%)	1640 (13.6%)	1762 (48.6%)
60–75 ml/min/1.73m^2^	977 (6.2%)	273 (2.3%)	704 (19.4%)
< 60 ml/min/1.73m^2^	200 (1.3%)	0	200 (5.5%)

*BMI* Body Mass Index, *SBP* Systolic Blood Pressure, *DBP* Diastolic Blood Pressure, *BUN*: Urea Nitrogen, *SCr* Serum Creatinine, *UA* serum Urine Acid, *LDL-C* Low-Density Lipoprotein Cholesterol, *FT3* Free Triiodothyronine, *FT4* Free Thyroxine, *TSH* Thyroid-Stimulating Hormone, *eGFR* estimated Glomerular Filtration Rate measured by the 2009 CKD-EPI formula

^*^ *P* < 0.05 compared to the younger adults

### Trends in thyroid function and eGFR with aging

**Fig. 1:**
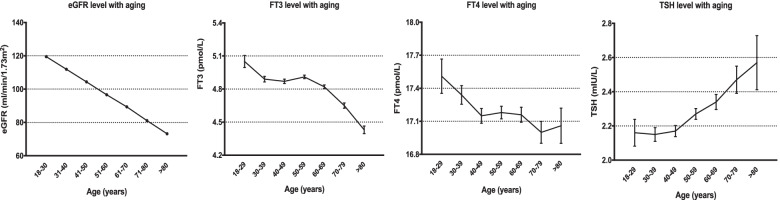
Trends of indicators of thyroid function and eGFR. FT3: free triiodothyronine, FT4: free thyroxine, TSH: thyroid-stimulating hormone, eGFR: estimated glomerular filtration rate by CKD-EPI formula

eGFR levels gradually decreased with aging. FT3 levels also decreased with aging (*P* for trend < 0.001) and decreased more rapidly in the older adults. FT4 tended to decline with age in those under 40 years of age, whereas it was relatively stable in those over 40 years of age. In addition, we observed an escalating trend of TSH level with aging in subjects older than 50 years of age (*P* for trend < 0.001) (Fig. 1).

### Association between thyroid function and eGFR

In older individuals, we verified a positive correlation between FT3 and eGFR (β = 0.083) after adjusting for gender, age, BMI, and UA. This correlation was also revealed in younger adults (β = 0.094). FT4 or TSH was negatively associated with eGFR in both subgroups. In the older population, TSH was more strongly correlated with eGFR (β = -0.081) than FT4 (β = -0.032), whereas the opposite was shown in the younger adults (β = -0.046 for TSH and β = -0.056 for FT4) (Table 2).

**Table 2:** Association between thyroid function and eGFR ^a^

	Younger Adults		Older Adults	
	β	*P* value	β	*P* value
FT3 (pmol/L)	0.094	< .001	0.083	< .001
FT4 (pmol/L)	-0.056	< .001	-0.032	0.032
TSH (mIU/L)	-0.046	< .001	-0.081	< .001

*eGFR* estimated Glomerular Filtration Rate calculated by the 2009 CKD-EPI formula, *FT3* Free Triiodothyronine, *FT4* Free Thyroxine, *TSH* Thyroid-Stimulating Hormone

^a^ Multiple linear regression analysis, adjusting for some traditional influencing factors like gender, age, body mass index, and serum urine acid

### Thyroid function and prevalence of reduced kidney function

The population with eGFR < 75 ml/min/1.73m^2^ were older, more likely to be male, and had higher UA. Lower FT3 and FT4 levels and significantly higher TSH levels were observed in older adults with eGFR < 75 ml/min/1.73m^2^ (Table 3). The incidence of reduced kidney function with different thyroid function was shown in Supplementary Table 1.

**Table 3:** Characteristics of the population with or without eGFR < 75 ml/min/1.73m^2^

Characteristic	Younger Adults		Older Adults	
	eGFR≧ 75 ml/min/1.73m^2^	eGFR < 75 ml/min/1.73m^2^	eGFR≧ 75 ml/min/1.73m^2^	eGFR < 75 ml/min/1.73m^2^
	(*N* 11,756)	(*N* 273)	(*N* 2724)	(*N* 900)
Female (*N*, %)	4873 (41.5%)	48 (17.6%) ^*^	893 (32.8%)	249 (27.7%) ^**^
Age (years)	48.46 (10.06)	57.31 (6.01) ^*^	72.09 (5.90)	76.9 (6.61) ^**^
BMI (kg/m^2^)	23.50 (2.35)	24.61 (1.96) ^*^	23.90 (2.23)	24.02 (2.30)
Hypertension (*N*, %)	3155 (26.8%)	122 (44.7%) ^*^	1888 (69.3%)	670 (74.4%)
Diabetes (*N*, %)	771 (6.6%)	10 (3.7%) ^*^	543 (19.9%)	145 (16.1%) ^**^
SCr (mg/dL)	0.78 (0.15)	1.11 (0.11) ^*^	0.78 (0.12)	1.03 (0.15) ^**^
UA (υmol/L)	333.89 (76.70)	404.86 (83.29) ^*^	335.67 (73.82)	385.32 (81.01) ^**^
FT3 (pmol/L)	4.89 (0.56)	4.88 (0.56)	4.70 (0.50)	4.54 (0.52)
FT4 (pmol/L)	17.20 (1.95)	17.27 (1.96)	17.10 (2.04)	17.05 (2.08)
TSH (mIU/L)	2.23 (1.07)	2.37 (0.85)	2.38 (1.42)	2.62 (2.01) ^**^
eGFR (ml/min/1.73m^2^)	102.05 (11.28)	70.33 (3.72) ^*^	87.04 (6.07)	65.57 (7.14) ^**^

*BMI* Body Mass Index, *SCr* Serum Creatinine, *UA* Urine Acid, *FT3* Free Triiodothyronine, *FT4* Free Thyroxine, *TSH* Thyroid- Stimulating Hormone, *eGFR* estimated Glomerular Filtration Rate measured by the 2009 CKD-EPI formula

^*^ *P* < 0.05 compared with the eGFR ≧75 ml/min/1.73m^2^ group in the younger subjects

^**^ *P* < 0.05 compared with the eGFR ≧75 ml/min/1.73m^2^ group in older subjects

**Fig. 2:**
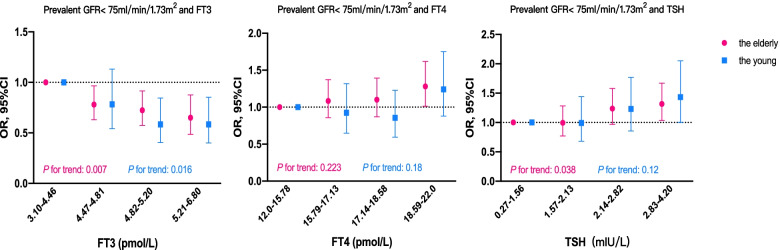
Association between thyroid function and prevalence of eGFR < 75 ml/min/1.73m^2^. FT3: free triiodothyronine, FT4: free thyroxine, TSH: thyroid-stimulating hormone, OR: odds ratio, 95%CI: 95% confidence interval

In the older adults, after adjustment for age, gender, BMI, and UA, the OR (95%CI) for eGFR < 75 ml/min/1.73m^2^ was 0.994 (0.77–1.283) among subjects with TSH 1.57–2.13 mIU/L, 1.236 (0.967–1.58) with TSH 2.14–2.82 mIU/L, and 1.315 (95%CI: 1.035–1.67) with TSH 2.83–4.20 mIU/L, comparing to individuals with TSH 0.27–1.56 mIU/L (*P* for trend: 0.038). On the contrary, the prevalence of eGFR < 75 ml/min/1.73m^2^ significantly decreased by 22.0% with FT3 4.47–4.81 pmol/L, 27.6% with FT3 4.82–5.20 pmol/L, and 34.9% with FT3 5.21–6.8 pmol/L compared with the population with the lowest quartile of FT3 (*P* for trend: 0.007). We found no significant evidence in correlation between FT4 and incident eGFR < 75 ml/min/1.73m^2^ in older people (*P* for trend: 0.223). In younger adults, the prevalence of eGFR < 75 ml/min/1.73m^2^ decreased with increasing FT3 levels within the reference range (*P* for trend: 0.016), but it was not significantly associated with FT4 and TSH (Fig. 2). Similar results were obtained in secondary analysis with further adjustment for hypertension and diabetes (Supplementary Table 2).

We also analyzed the correlation between the prevalence of eGFR < 60 ml/min/1.73m^2^ and thyroid function in the older population. The prevalence of eGFR < 60 ml/min/1.73m^2^ significantly decreased by 41.8% among individuals with FT3 4.47–4.81 pmol/L and 69.9% with FT3 5.21–6.8 pmol/L compared to the older population with FT3 3.10–4.46 pmol/L (*P* for trend: 0.002). Additionally, their prevalence increased with elevating FT4 and TSH levels, which was not statistically significant.

## Discussion

In older adults, eGFR levels were significantly negatively correlated with TSH but significantly positively correlated with FT3. The risk of reduced kidney function was higher in subjects with low-normal FT3 and high-normal TSH levels. The prevalence of reduced kidney function was not associated with FT4 within the reference range.

We investigated a rapid decrease in FT3 with aging in the older population. This trend may be due to decrease in the activity of 5’mono-deiodase catalyzing deiodination of T4 into T3 or/and reduced stimulation by TSH with aging [8]. The trend in FT4 levels in the older population is consistent with previous studies, which also demonstrated little change of FT4 concentrations with aging [13]. This stability despite aging may be attributed to reduction of synthesis and secretion, but a decrease in metabolic thyroxine clearance [8]. This study also observed that TSH levels increased with age based on this population. The trends in thyroid function and kidney function in this population are consistent with previous studies; thus, the subjects in this study were well represented.

The risk of eGFR < 75 ml/min/1.73m^2^ or eGFR < 60 ml/min/1.73m^2^ both gradually decline with increasing FT3 within the reference range. Decreased FT3 levels have been shown to be related to endothelial dysfunction and inflammation, which are pathology of aged and impaired kidney [14]. Several studies have shown that the prevalence of eGFR < 60 ml/min/1.73m^2^ was significantly greater in the low T3 condition [15, 16]. Several studies have demonstrated the association between the presence of low T3 condition and acceleration of disease progression and some adverse outcomes in patients with heart disease or surgery therapy in older adults [15–17]. To date, studies and guidelines concentrated on the cardio and mental prognosis in patients with low T3, but rare of them focused on the kidney prognosis [8, 18]. Meanwhile, Kidney is crucial for the metabolism and excretion of thyroid hormones, and low serum T3 is a common thyroid hormone disturbance during illness, including impaired kidney function [14, 18]. Peters et al. demonstrated that the prevalence of low T3 syndrome was 2.5 times higher in patients with advanced kidney disease than those with normal kidney function [19]. Therefore, our study revealed that low FT3 levels might be associated with the incidence of decreased kidney function in the older population even within the reference range. In addition, FT3 may be associated with age-related pathology in kidney.

A significant association was investigated between TSH and eGFR or risk of reduced kidney function in older people. The prospective Kangbuk Samsung Health Study has shown that individuals with high-normal levels of TSH had an increased risk of reduced kidney function [12]. Besides, A study from the Netherlands showed a similar result in older population (age ≧85 years) [11]. Conversely, several studies based on the general population demonstrated that TSH was not associated with the risk of kidney function decline [10, 12, 20, 21]. Differences in race, age, and included correction factors may have contributed to these conflicting results. Therefore, considering the application of TSH as an essential diagnostic and efficacy evaluation indicator in current clinical applications, further stuides are needed on the association between TSH and reduced kidney function to verify whether TSH is an appropriate indicator for follow-up observation of older adults with declining kidney function.

FT4 concentrations leveled off in the older population. No significant association was found in older adults between FT4 and the prevalence of reduced kidney function, which is consistent with previous studies [21, 22]. Meanwhile, some studies demonstrated positive or negative correlation between FT4 and kidney function [11, 12]. There was no significant association between normal-range FT4 and prevalence of eGFR < 75 ml/min/1.73m^2^ in younger adults.

Thyroid function may influence kidney function through several mechanisms. Elevated thyroid function may improve kidney function by regulating renal blood flow, renin–angiotensin–aldosterone system, nitric oxide synthase activity, and sodium reabsorption [8]. However, several studies recently verified that elevated thyroid hormone levels could enhance oxygen consumption and production of reactive oxygen species (ROS), which may subsequently induce DNA damage and cell apoptosis [23, 24]. Oxidative stress, caused by the increased production of ROS, is a pivotal factor of impaired kidney function through glomerular damage, renal ischemia, inflammation, solute and water reabsorption, and endothelial dysfunction [25, 26]. It has also been suggested that hypothyroidism in older adults represents a physiological downregulation of the hypothalamic-pituitary axis, possibly benefitting life expectancy [27, 28]. In addition, a possible explanation lies in a slower metabolic rate relating to increased survival [29]. Meanwhile, impaired kidney function is a state of high oxidative stress, inflammation, and malnutrition, conditions favoring low-T3 levels [30, 31]. The mechanisms are complex between thyroid and kidney function, so further studies are needed to clarify and may provide a new perspective on the development of age-relate decline in kidney function.

The data presented here could be promising for clinical applications in older adults. The evaluation of thyroid function is needed in older patients with pathologically declining in kidney function. Meanwhile, for patients with potentially pathologic declines in renal function, clinicians need to access kidney function in both inpatients on initial visit and outpatient receiving follow-up with low-normal FT3 or high-normal TSH level. Furthermore, FT3 may be a better index to evaluate the clinical curative effect of patients with thyroid function disorder and renal insufficiency according to the association between FT3, FT4, and TSH and prevalence of reduced kidney function. Additionally, considering the change of thyroid function with aging and prognosis of related organs, the reference intervals of thyroid hormones in older adults need to be further discussed.

Our study is based on a large check-up Chinese population within a real-world clinical environment, discussing the association between thyroid function and kidney function in older adults and observing the difference between the younger and older adults. In addition, we performed adjustments for several identified and potential confounders. As an identified risk factor for decreased renal function, UA was not included in the calibration in previous studies. Most importantly, the association between normal FT3 levels and kidney function was first analyzed in detail in the older Chinese population.

This study has some limitations that may influence our results. First, we estimated GFR by the CKD-EPI formula based on SCr rather than the golden standard. eGFR may not be a good approximation of kidney function in this association as thyroid function could influence SCr levels via muscle metabolism and volume status [32]. However, our previous research investigated the high accuracy of the CKD-EPI formula in older Chinese adults. We also observed the correlation between thyroid function and eGFR by FAS formula, which performed well in healthy older people and obtained similar results. In addition, several studies have demonstrated that the incidence of thyroid disease was significantly elevated in the population with impaired kidney function [19, 33]. It is still unclear whether thyroid hormone alterations are a cause or a consequence of reduced kidney function. Further studies are required to elucidate the causal relationship between thyroid function and kidney function.

## Conclusions

In conclusion, we demonstrated a significant association between normal-range thyroid function and age-related decline in kidney function, particularly FT3 levels in older adults. Clinicians may need to pay more attention to the assessment and follow-up of kidney function in older individuals with low-normal FT3 and high-normal TSH. Further cohort studies are needed to investigate the causal relationship and mechanisms between thyroid function and kidney function, and to detect an adaptive method and timing of interventions and proper reference range to get a better kidney prognosis in the older population.

## Supplementary Information

**Additional file 1:** **Supplementary Table 1**. Incidence of reduced kidney function with different thyroid function.

**Additional file 2:** **Supplementary Table 2.** Association between thyroid function and prevalence of eGFR < 75ml/min/1.73m^2^^*^_._

## Data Availability

The datasets used and analyzed during the current study are available from the corresponding author on reasonable request.

## Abbreviations

eGFR: Estimated glomerular filtration rate
CKD: Chronic kidney disease
SCr: Serum creatinine
TSH: Thyroid-stimulating hormone
FT4: Free thyroxine
FT3: Free triiodothyronine
BMI: Body mass index
BUN: Urea nitrogen
UA: Serum urine acid
CKD-EPI: Chronic Kidney Disease Epidemiology Collaboration
BG: Blood glucose
ALT: Alanine transaminase
LDL-C: Low-density lipoprotein cholesterol
