# Supplementary material for: Thyroid function and age-related decline in kidney function in older Chinese adults: a cross-sectional study
Source: BMC Geriatr. 2022 Mar 17;22:221. doi: 10.1186/s12877-022-02904-z (PMC8932124; doi:10.1186/s12877-022-02904-z)
Supplement: Supplementary file 1 — Additional file 1: Supplementary Table 1. Incidence of reduced kidney function with different thyroid function. [file 12877_2022_2904_MOESM1_ESM.docx]

**Supplementary Table 1**. Incidence of reduced kidney function with different thyroid function

|  | eGFR< 75ml/min/1.73m^2^ (N/N) | | eGFR< 60ml/min/1.73m^2^ (N/N) | |
| --- | --- | --- | --- | --- |
|  | Younger Adults | Older Adults | Younger Adults | Older Adults |
| FT3 (pmol/L) |  |  |  |  |
| 3.10-4.46 | 62/2686 | 402/1292 | - | 110/1292 |
| 4.47-4.81 | 67/2860 | 229/995 | - | 39/995 |
| 4.82-5.20 | 72/3155 | 181/824 | - | 38/824 |
| 5.21-6.8 | 72/3328 | 88/513 | - | 8/513 |
| FT4 (pmol/L) |  |  |  |  |
| 12.0-15.78 | 70/2928 | 253/1006 | - | 59/1006 |
| 15.79-17.13 | 65/3046 | 212/866 | - | 44/866 |
| 17.14-18.58 | 59/3042 | 203/862 | - | 43/862 |
| 18.59-22.0 | 79/3013 | 232/890 | - | 49/890 |
| TSH (mIU/L) |  |  |  |  |
| 0.27-1.56 | 57/3102 | 186/823 | - | 38/823 |
| 1.57-2.13 | 62/3084 | 188/824 | - | 35/824 |
| 2.14-2.82 | 73/2976 | 239/939 | - | 50/939 |
| 2.83-4.20 | 81/2867 | 287/1038 | - | 72/1038 |

FT3: free triiodothyronine, FT4: free thyroxine, TSH: thyroid-stimulating hormone
