# Supplementary material for: Thyroid function and age-related decline in kidney function in older Chinese adults: a cross-sectional study
Source: BMC Geriatr. 2022 Mar 17;22:221. doi: 10.1186/s12877-022-02904-z (PMC8932124; doi:10.1186/s12877-022-02904-z)
Supplement: Supplementary file 2 — Additional file 2: Supplementary Table 2. Association between thyroid function and prevalence of eGFR < 75ml/min/1.73m2*. [file 12877_2022_2904_MOESM2_ESM.docx]

Supplementary Table 2. Association between thyroid function and Prevalence of eGFR < 75ml/min/1.73m^2^ ^*^

|  | Younger Adults | | | Older Adults | | |
| --- | --- | --- | --- | --- | --- | --- |
|  | OR | 95%CI | *P* value | OR | 95%CI | *P* value |
| FT3 (pmol/L) |  |  |  |  |  |  |
| 3.10-4.46 | Reference |  |  | Reference |  |  |
| 4.47-4.81 | 0.783 | 0.542-1.132 | 0.193 | 0.769 | 0.622-0.951 | 0.016 |
| 4.82-5.20 | 0.585 | 0.404-0.846 | 0.004 | 0.709 | 0.561-0.895 | 0.004 |
| 5.21-6.80 | 0.584 | 0.400-0.853 | 0.005 | 0.631 | 0.469-0.849 | 0.002 |
| FT4 (pmol/L) |  |  |  |  |  |  |
| 12.0-15.78 | Reference |  |  | Reference |  |  |
| 15.79-17.13 | 0.934 | 0.654-1.333 | 0.706 | 1.093 | 0.863-1.383 | 0.462 |
| 17.14-18.58 | 0.877 | 0.609-1.265 | 0.483 | 1.115 | 0.88-1.413 | 0.369 |
| 18.59-22.0 | 1.293 | 0.914-1.828 | 0.146 | 1.301 | 1.028-1.646 | 0.028 |
| TSH (mIU/L) |  |  |  |  |  |  |
| 0.27-1.56 | Reference |  |  | Reference |  |  |
| 1.57-2.13 | 0.999 | 0.686-1.454 | 0.994 | 0.982 | 0.76-1.269 | 0.892 |
| 2.14-2.82 | 1.227 | 0.853-1.765 | 0.27 | 1.216 | 0.951-1.556 | 0.119 |
| 2.83-4.20 | 1.45 | 1.012-2.079 | 0.043 | 1.301 | 1.023-1.654 | 0.032 |

^*^ Adjustment for age, gender, BMI, UA, hypertension, and diabetes.

FT3: free triiodothyronine, FT4: free thyroxine, TSH: thyroid-stimulating hormone, OR: odds ratio, 95%CI: 95% confidence interval.
